# *In silico* characterization of aryl benzoyl hydrazide derivatives as potential inhibitors of RdRp enzyme of H5N1 influenza virus

**DOI:** 10.3389/fphar.2022.1004255

**Published:** 2022-09-26

**Authors:** Abhishek Ghosh, Parthasarathi Panda, Amit Kumar Halder, Maria Natalia D. S. Cordeiro

**Affiliations:** ^1^ Dr. B. C. Roy College of Pharmacy and Allied Health Sciences, Durgapur, West Bengal, India; ^2^ LAQV@REQUIMTE/Department of Chemistry and Biochemistry, Faculty of Sciences, University of Porto, Porto, Portugal

**Keywords:** influenza virus, RNA-dependent RNA polymerase, QSAR, pharmacophore mapping, molecular dynamics, *in silico*, antiviral agents

## Abstract

RNA-dependent RNA polymerase (RdRp) is a potential therapeutic target for the discovery of novel antiviral agents for the treatment of life-threatening infections caused by newly emerged strains of the influenza virus. Being one of the most conserved enzymes among RNA viruses, RdRp and its inhibitors require further investigations to design novel antiviral agents. In this work, we systematically investigated the structural requirements for antiviral properties of some recently reported aryl benzoyl hydrazide derivatives through a range of *in silico* tools such as 2D-quantitative structure-activity relationship (2D-QSAR), 3D-QSAR, structure-based pharmacophore modeling, molecular docking and molecular dynamics simulations. The 2D-QSAR models developed in the current work achieved high statistical reliability and simultaneously afforded in-depth mechanistic interpretability towards structural requirements. The structure-based pharmacophore model developed with the docked conformation of one of the most potent compounds with the RdRp protein of H5N1 influenza strain was utilized for developing a 3D-QSAR model with satisfactory statistical quality validating both the docking and the pharmacophore modeling methodologies performed in this work. However, it is the atom-based alignment of the compounds that afforded the most statistically reliable 3D-QSAR model, the results of which provided mechanistic interpretations consistent with the 2D-QSAR results. Additionally, molecular dynamics simulations performed with the apoprotein as well as the docked complex of RdRp revealed the dynamic stability of the ligand at the proposed binding site of the receptor. At the same time, it also supported the mechanistic interpretations drawn from 2D-, 3D-QSAR and pharmacophore modeling. The present study, performed mostly with open-source tools and webservers, returns important guidelines for research aimed at the future design and development of novel anti-viral agents against various RNA viruses like influenza virus, human immunodeficiency virus-1, hepatitis C virus, corona virus, and so forth.

## Introduction

Influenza is a severe infectious respiratory disease caused in humans mainly by influenza A and influenza B viruses, though other strains like influenza C and D are also identified (Krammer et al., 2018; Javanian et al., 2021). Influenza A virus is mainly responsible for sporadic pandemic outbreaks and is regarded as the most the deadliest strain of influenza due to its antigenic shift and drift, whereas both influenza A and B may cause an epidemic or seasonal influenza (Ziegler et al., 2018). Symptoms of influenza may vary from mild upper respiratory infection (characterized by fever, soar throat, runny nose, muscle pain and fatigue) to lethal pneumonia (Jochems et al., 2018). Apart from lung, influenza infection may also damage the cardiovascular, muscular, central nervous system, and other organ systems. Furthermore, highly pathogenic influenza strains such as H5N1 and H7N9 have emerged in recent years with considerably higher transmission and mortality rates (Krammer et al., 2018; Sherman et al., 2019). Similar to other RNA viruses more deadly variants of influenza A may emerge in near future (Su et al., 2017). As far as therapeutic options are concerned, three major classes of anti-influenza drugs are available and these include *1*) M2 proton channel inhibitors, *2*) neuraminidase inhibitors and *3*) polymerase basic protein 1 inhibitors (De Clercq, 2006; Principi et al., 2019). However, drug-resistant mutations have been frequently reported both in M2 and neuraminidase enzymes restricting the therapeutic potential of the inhibitors of these proteins (Samson et al., 2013; Digard et al., 2015; Principi et al., 2019). Therefore, to improve the availability of therapeutic options for highly resistant influenza strains, it is imperative that new therapeutic targets of influenza are investigated properly in the search for novel anti-influenza agents. RNA-dependent RNA polymerase (RdRp) is one of such potential therapeutic targets that is highly conserved among various strains of influenza A as well as among other RNA viruses like zika viruses, coronaviruses, hepatitis C virus, dengue virus, norovirus, and measles (Stubbs and te Velthuis, 2014; Venkataraman et al., 2018). RdRp is a vital enzyme for the viral replication process, catalyzing the viral RNA template-dependent development of phosphodiester bonds using a metal ion-dependent mechanism (Picarazzi et al., 2020; Tian et al., 2021). The RdRp is a heterotrimer composed of three covalently-bound subunits, namely: PA − endonuclease subunit polymerase acidic protein, PB1 − polymerase catalytic subunit polymerase basic protein 1, and PB2 − cap-binding subunit polymerase basic protein 2 (Massari et al., 2016; Ren et al., 2021). Compounds disrupting the function of any of these subunits or blocking the interactions between any two subunits may therefore function as potential lead molecules against influenza A infection. Indeed, two compounds namely favipiravir and Xofluza (baloxavir marboxil) have been approved for influenza treatment (Nagata et al., 2014; Takashita et al., 2016). Nevertheless, teratogenicity was reported with favipiravir treatment whereas Xofluza, being a PA subunit inhibitor, is susceptible to bring about a mutation (I38/T/M) leading towards significant reduction of its efficacy (Nagata et al., 2014; Agrawal et al., 2020). Therefore, new RdRp inhibitors acting on subunits other than PA, such as PB1, should be studied thoroughly to obtain novel lead molecules against influenza A. In a recent study, Liu et al. reported a series of aryl benzoyl hydrazide derivatives as RNA-dependent RNA polymerase inhibitors of influenza A (Liu et al., 2022). Furthermore, preliminary mechanistic studies conducted by the same authors suggested that these compounds may exhibit anti-influenza virus activity by binding to the PB1 subunit of RdRp (Liu et al., 2022). Besides, recent works showed that RdRp inhibitors are promising potent anti-RNA virus drugs that may ultimately be used for the treatment of the coronavirus disease 2019 (COVID-19) (Pachetti et al., 2020; Picarazzi et al., 2020; Dejmek et al., 2021; Tian et al., 2021).

Ligand- and structure-based *in silico* modelling strategies have been successfully employed in the past to design and develop novel therapeutic agents (Sabe et al., 2021). In the present work, we report cheminformatic modeling approaches such as 2D and 3D-quantitative structure-activity relationships (2D-QSAR and 3D-QSAR) to characterize the structural requirements of the aryl benzoyl hydrazide derivatives reported by Liu et al. (Liu et al., 2022) for owning higher potency against influenza A. In the later study, the synthesized compounds were primarily assayed against avian H5N1 flu strain with 50% effective concentration (EC_50_) values ranging from 9.3 nM to 86 μM. Clearly, such a large range of biology activity for any focused library developed with structurally similar compounds demands detailed *in silico* investigations to identify crucial structural attributes for higher biological potency. That is especially important since the work of Liu et al. provided only the molecular docking of one of the most potent ligands of this dataset and lacked a detailed structure-activity relationship to explain the large variations in the biological activity obtained for the compounds. Apart from these ligand-based approaches, this work also includes the development of a structure-based pharmacophore model and it investigates both the binding potential and the dynamic behavior of these compounds against RdRp enzyme through molecular dynamics (MD) simulations.

## Materials and methods

### Dataset collection and structure preparation

Thirty aryl benzoyl hydrazide derivatives were collected from the recently published work by Liu et al. (Liu et al., 2022). The anti-influenza activity of these compounds was reported against the H5N1 strain and the cellular assays were performed in MDCK cells by phenotypic cell protection (CPE) using the cell counting kit (CCK-8) method. The 50% effective concentration (EC_50_ in µM) values of these compounds were log-transformed (pEC_50_ = −log_10_ (EC_50_/10^6^) for subsequently use as the response variables in both the 2D- and 3D-QSAR modeling. The structures and biological activity of these compounds are provided in the Supplementary Table S1. Note that we did not alter the numbering of the dataset compounds provided by Liu et al. (Liu et al., 2022). It is also worth mentioning here that, even though these compounds are structurally similar, according to the similarity analysis conducted using the SIMSEARCH tool (Halder and Cordeiro, 2021a; the results of this analysis can be found in the supplementary materials, Supplementary Text S1), a long range of biological activity (with around four log unit differences) was noted further justifying their thorough *in silico* investigation. The SMILES notation of these structures provided by Liu et al. (Liu et al., 2022) was converted to.sdf file using Discovery Studio Visualization tool and then numbered accordingly. These structures were then standardized using the Standardizer tool of Chemaxon using the following options: *1*) add explicit hydrogen atoms, *2*) aromatize, *3*) clean 2D, *4*) clean 3D, *5*) neutralize and *6*) strip salts. The standardized structures were further processed for the 2D- and 3D-QSAR modeling studies (ChemAxon, 2010).

### 2D-QSAR modeling

#### Descriptor calculation

Descriptors for the thirty aryl benzoyl hydrazide derivatives were calculated using alvaDescv.2.0.4 (https://www.alvascience.com/alvadesc/) (Mauri, 2020) under the OCHEM webserver (Sushko et al., 2011). For the calculation of 3D descriptors, the structures of the compounds were geometrically optimized in this web platform using the Corina tool (Sadowski et al., 2002). The calculated descriptors of these compounds were then merged with the respective pEC_50_ of the compounds to form the dataset for 2D-QSAR model generation.

#### Dataset division and model development

The dataset was divided into a training and a test set by the activity sorting method using the Python based open-access SFS-QSAR tool (https://github.com/ncordeirfcup/SFS-QSAR-tool) (Halder et al., 2022) with starting point 2. The models were developed in three stages. In the first stage, some selected descriptors having higher overall interpretability were considered in search for interpretable 2D-QSAR models. These descriptors belong to the categories of constitutional descriptors, functional group counts, 2D-atom pairs, drug-like indices, ring descriptors, atom-centered fragments, pharmacophore descriptors and molecular properties. In the second stage, only 2D descriptors were applied for model generation and finally, in the last stage all types of descriptors were employed for model generation. The purpose here was to understand whether the inclusion of 2D descriptors improved the quality of the model to a considerable extent or not. Similarly, in the third stage, we try to access whether 3D descriptors, the values of which are sensitive to the specific 3D conformation, are absolutely essential for characterizing the structural requirement of the compounds or not. 2D-QSAR models were developed using two feature selection algorithms, *i.e*.: *1*) sequential forward selection (SFS) and *2*) the genetic algorithm (GA). The SFS based model was developed using the newly developed open-access SFS-QSAR tool (https://github.com/ncordeirfcup/SFS-QSAR-tool) which resorts to the “Feature Selector” module of the library Mlxtend (http://rasbt.github.io/mlxtend/) (Halder et al., 2022). Data treatment was performed by setting a variance cut-off of 0.0001 to remove constant and near-constant descriptors, and a correlation cut-off of 0.99 to eliminate highly inter-correlated descriptors. Even though a high correlation cut-off was chosen, the cross-correlation matrix of each model was examined to check for the presence of highly intercorrelated descriptors. In the case of finding highly correlated descriptors, the correlation cut-off was thereafter reduced. During model development, four scoring functions of the “Sequential Feature Selector” module were employed for feature selection, namely: the determination coefficient (*R* ^2^), the negative mean absolute error (NMAE), the negative mean Poisson deviance (NMPD), and the negative mean gamma deviance (NMGD). No cross-validation was carried out during feature selection. GA-based models were set-up by resorting to the open access tool GeneticAlgorithm v.4.1_2 (accessed from https://dtclab.webs.com/software-tools) (Ambure et al., 2015). In contrast to SFS, which is considered a non-stochastic feature selection method, GA follows stochastic algorithms to generate randomized models and it employs tools such as cross-over and mutations to improve the fitting of the independent variables with the response variable. Similar to the SFS-QSAR modeling, a descriptor pre-treatment was carried out during development of the GA-based models. A maximum of five descriptors were initially allowed in the 2D-QSAR models and after confirming the best model, it was checked whether the model truly requires five descriptors. The latter was assessed using the SFS-QSAR tool by setting the “% of CV increment” to 5. In so doing, the models were regenerated with the condition that a descriptor is included in the model only if its inclusion increases the leave-one-out (LOO) cross-validated regression coefficient (*Q* ^2^_LOO_) at least 5% with respect to the existing model.

#### Statistical analysis of the models

The goodness of fit, robustness, and internal predictivity of the final 2D-QSAR models were checked by a range of well-known statistical metrics. Both the coefficient of determination, *R* ^2^, the adjusted *R* ^2^ (*R* ^2^_Adj_), the Fisher’s statistics (*F*-test), and the mean absolute error (MAE) were used to measure the goodness of fit of the models, whereas the internal cross-validation coefficient *Q* ^2^_LOO_ (leave-one-out) was used to check their robustness and internal predictivity (Tetko et al., 2001; Halder, 2018). The external validation metric *R* ^2^_Pred_ was employed to judge the external predictivity of the models (Golbraikh and Tropsha, 2002). Apart from these, *r*_*m*_^2^ metrics such as *r*_*m*_^2^_ (LOO)_ and *∆r*_*m*_^2^_  (LOO)_ were used as internal validation parameters, while *r*_*m*_^2^_  (test)_ and *∆r*_*m*_^2^_ (test)_ were used as external validation parameters (Roy et al., 2009). The best QSAR model was also checked for inter-collinearity among its descriptors. Furthermore, the *Y*-randomization test was repeatedly run to generate 1,000 models with randomized response variables to check for chance correlations, and the parameter ^*c*^ *R*_*p*_^2^ calculated. A higher value for ^*c*^ *R*_*p*_^2^ implies that the original model was not developed by chance (Ojha and Roy, 2011).

#### Applicability domain of the models

In this work, the applicability domain of the models was estimated by resorting to the so-called William’s plot, which is a plot drawn between the leverage values and standardized coefficients. If the leverage value of any data-point is larger than the hat value h* (h* = 3*p’*/*n*, where *p’* is number of model’s descriptors + 1 and *n* is the number of data-points in the training set), it is considered as a structural outlier. By contrast, if the standardized residual of the data-point is > ±2.5, it is considered as a response outlier (Roy et al., 2015).

### 3D-QSAR modeling

#### Alignment methods

The following two types of alignment methods were used in the current work, namely: *1*) atom-based alignment or unsupervised rigid-body molecular alignment (Tosco et al., 2011) and *2*) supervised alignment based on the structure based pharmacophore model. For the rigid body molecular alignment, the 3D structures of the ligands were first minimized using the “obminimize” function of OpenBabel tool and the steepest descent method. The minimized structures were subsequently submitted to generate 100 conformations followed by alignment with the help of *rdMolAlign.GetCrippenO3A* program of Rdkit. The Python scripts used for atom-based alignment are provided in the Github repository https://github.com/ncordeirfcup/InsilicoModeling_RdRp (i.e., *alignment.py*). For the structure-based pharmacophore modeling, the docked structure of one of the most potent ligands with RdRp protein was utilized and the *StructureBasedPharmacophore* feature of the newly developed OpenPharmacophore (https://github.com/uibcdf/OpenPharmacophore) was employed using the options: “radius” as 1.0 and hydrophobic as “plip.” The developed pharmacophore model was then used for screening of all dataset molecules using both the *EmbedLib.MatchPharmacophore* and *EmbedLib.EmbedPharmacophore* functions of Rdkit. The Jupyter notebook files used for setting up the structure-based pharmacophore model and for screening of ligands with the later model can be found in the Github repository https://github.com/ncordeirfcup/InsilicoModeling_RdRp (*i.e.*, files *strbased_pharmacophore_development.ipynb* and *strbased_pharmacophore_screen.ipynb*). The structure-based pharmacophore model was developed without any modification of the code of Openpharmacophore, as shown in the file *strbased_pharmacophore_development.ipynb*.

#### Model development

3D-QSAR modeling was performed with the open-source software named Open3DQSAR (Tosco and Balle, 2010). The detailed methodologies of this tool have been discussed earlier (Tosco and Balle, 2010). Briefly, this software uses a carbon and volume-less positively (+1) charged probe for calculating steric and electrostatic fields of the query chemicals, respectively. The pre-treatment of the fields was carried out after setting a smart region definition (SRD) cut-off level of 2.0 and also by removing *N*-level variables. The Open3DQSAR uses SRD for variable grouping, based on the closeness of variables in 3D space, as well as two different variable selection algorithms, namely: Fractional Factorial Design-based variable SELection (FFD-SEL), and the Uninformative Variable Elimination-based Partial Least Square (UVE-PLS). The predictive quality of the 3D-QSAR-based PLS models generated from the compounds was examined using the coefficient *R* ^2^ with its standardized errors of calibration (SDEC), *F*-test results, *Q* ^2^_LOO_, leave-two-out *Q* ^2^ (*Q* ^2^_LTO_), leave-many-out *Q* ^2^ (*Q* ^2^_LMO_, with five groups, and 20 runs), and *R* ^*2*^_Pred_ with its associated standardized errors of prediction (SDEP) values. Additionally, to check if the model was unique and not developed by chance, progressive scrambling methods were run for the selected models by applying the following criteria: critical value: 0.80, type: LMO groups = 5, runs = 20, and scrambling = 20. The output of the scrambling test appeared as “fitted q2 values” in Open3DQSAR and is denoted as *Q* ^2^_s_ in the current work. The low value of this parameter as compared to *Q* ^2^_LMO_ justifies the robustness of the generated 3D-QSAR model. The contour maps were visualized with isocontour values at PLS coefficients of +0.005 (green) and −0.005 (yellow) for the steric filed and +0.003 (blue) and −0.003 (red) for the electrostatic field. All plots for the 2D-QSAR and 3D-QSAR models were generated using the matplotlib software.

### Molecular docking analysis

The semi-rigid molecular docking of the selected dataset compounds was conducted with the crystal structure of RdRp (PDB: 6QPF) (Fan et al., 2019) collected from the Protein Data Bank (https://www.rcsb.org/) (Berman et al., 2003). The B-chain of this crystal structure that represents the PB1 domain of the protein (Liu et al., 2022) was selected from the docking analysis with AutoDock Vina (version 1.1.2., The Scripps Research Institute, La Jolla, CA, United States) (Trott and Olson, 2010). To begin with, a blind docking was performed using a grid box center located at the center of the macromolecule and grid box dimension as 126 × 126 × 126 Å^3^. After locating the possible binding site from this blind docking, a grid box of 40 × 40 × 40 Å^3^ dimension was located at *X* = 61.1, *Y* = 1.9 and *Z* = 7.1. The missing chains of the protein were included using the Modeller program (Eswar et al., 2014) under the Chimera platform (Pettersen et al., 2004). The protein *pdbqt* file was prepared by removing the water molecules, and adding hydrogen atoms and Gasteiger–Marsili partial atomic charges (Halder et al., 2019). The energy minimized structures of the ligands as described in the previous section were converted to *pdbqt* forms and these were subsequently submitted for Audock Vina-based docking with an exhaustiveness equal to 45.

### Molecular dynamics simulations

MD simulations were carried out using the software package AMBER 20 (D.A. Case et al., 2021). The PDB2PQR server (http://server.poissonboltzmann.org/pdb2pqr) (Dolinsky et al., 2007; Bas et al., 2008) was utilized to fix the protonation states of the amino acid residues of each protein (at pH = 7.0) using the AMBER forcefield and output naming scheme. The ff99SB and general AMBER forcefield (GAFF) were employed to describe the protein and inhibitor interactions, respectively. The ligand parameterizations for the protein complex were performed with the Leap program using GAFF in the Antechamber (Wang et al., 2004; Hornak et al., 2006). MD simulations were performed with a TIP3P cubic box (Jorgensen et al., 1983) set with 8 Å distance around the apoprotein or protein complex. The positive charges of the apoprotein and protein complex were neutralized by adding chloride ions. The Partial Mesh Ewald (PME) method was considered for the long-range electrostatic forces with a cut-off of 12 Å (Halder and Honarparvar, 2019). The SHAKE algorithm was used to constrain all bonds involving hydrogen atoms. The energy minimization of the system was performed in two steps. First, only ions and water molecules were minimized within a 2000 step minimization process (1,000 steps of steepest decent minimization followed by 1,000 of conjugated gradient) using a restrained force of 500 kcal/mol on the solute. Second, the whole apoprotein or protein complex was relaxed by employing a 5000 step minimization process (2,500 steps of steepest decent minimization followed by 2,500 of conjugated gradient). The minimized system was heated up stepwise from 0 to 300 K with a weak harmonic restraint of 10 kcal/mol keeping the solute fixed for 200 ps. Then, a constant pressure equilibration at 300 K followed for 2 ns. Finally, MD simulations without any restriction were run for 30 ns keeping constant the temperature (300 K) and pressure (1 atm) (Halder and Honarparvar, 2019).

After completion of simulation, post-dynamics analysis over the MD trajectories was performed using the PTRAJ and CPPTRAJ modules implemented in AMBER to analyze the root mean square deviation (RMSD), root mean square fluctuation (RMSF) and average structure of the complex (Roe and Cheatham, 2013). Furthermore, the molecular mechanics generalized born surface area (MM-GBSA) (Srinivasan et al., 1998) binding free energies of the protein-ligand complexes were calculated using the *MM-PBSA.py* tool of Amber and by employing 100 snapshots taken from the last 10 ns of the MD trajectory. A detailed and in-depth description and discussion of the MM-GBSA analysis applied here can be found in our recent work (Halder and Cordeiro, 2021b). However, the entropic contribution (T∆S) is not calculated since it is computationally expensive for large protein complexes and its accuracy may not always be ascertained (Berishvili et al., 2020; Halder and Cordeiro, 2021b).

The energy contributions of the binding site amino acid residues into the total binding free energies were computed using the MM-GBSA per residue free energy decomposition method with Amber MM-GBSA module (Srinivasan et al., 1998; Genheden and Ryde, 2015; Halder and Honarparvar, 2019). All energy components (van der Waals, electrostatic, polar solvation, and nonpolar solvation contributions) were calculated using 200 snapshots extracted from the last 10 ns MD trajectories.

## Results and discussions

### 2D-QSAR modeling

Two different methods namely SFS-MLR and GA-MLR were applied separately for setting up 2D-QSAR linear models from a set of interpretable descriptors, as well as 0D-2D and 0D-3D descriptors. A summary of the obtained statistical results is presented in Table 1.

**TABLE 1:** Summary of the results obtained from 2D-QSAR modeling performed with the selected interpretable descriptors, 0D-2D and all 0D-3D descriptors ^a^.

Method	Interpretable descriptors (*n* = 3139)			0D-2D descriptors (*n* = 1904)			0D-3D descriptors (*n* = 417)		
	Scoring	*Q* ^2^_LOO_	*R* ^2^_Pred_	Scoring	*Q* ^2^_LOO_	*R* ^2^_Pred_	Scoring	*Q* ^2^_LOO_	*R* ^2^_Pred_
SFS-MLR	*R* ^2^	0.78	0.586	*R* ^2^	0.834	0.733	*R* ^2^	0.886	0.889
	NMAE	0.742	0.726	NMAE	0.781	0.706	NMAE	0.830	0.c911
	NMPD	0.560	0.314	NMPD	0.834	0.733	NMPD	0.886	0.889
	NMGD	0.560	0.314	NMGD	0.807	0.693	NMGD	0.886	0.889
GA-MLR	—	**0.843**	**0.842**	—	**0.839**	**0.925**	—	**0.865**	**0.925**

a The number of descriptors used for model development are shown inside parenthesis. The best QSAR models found are highlighted in bold.

**FIGURE 1:**
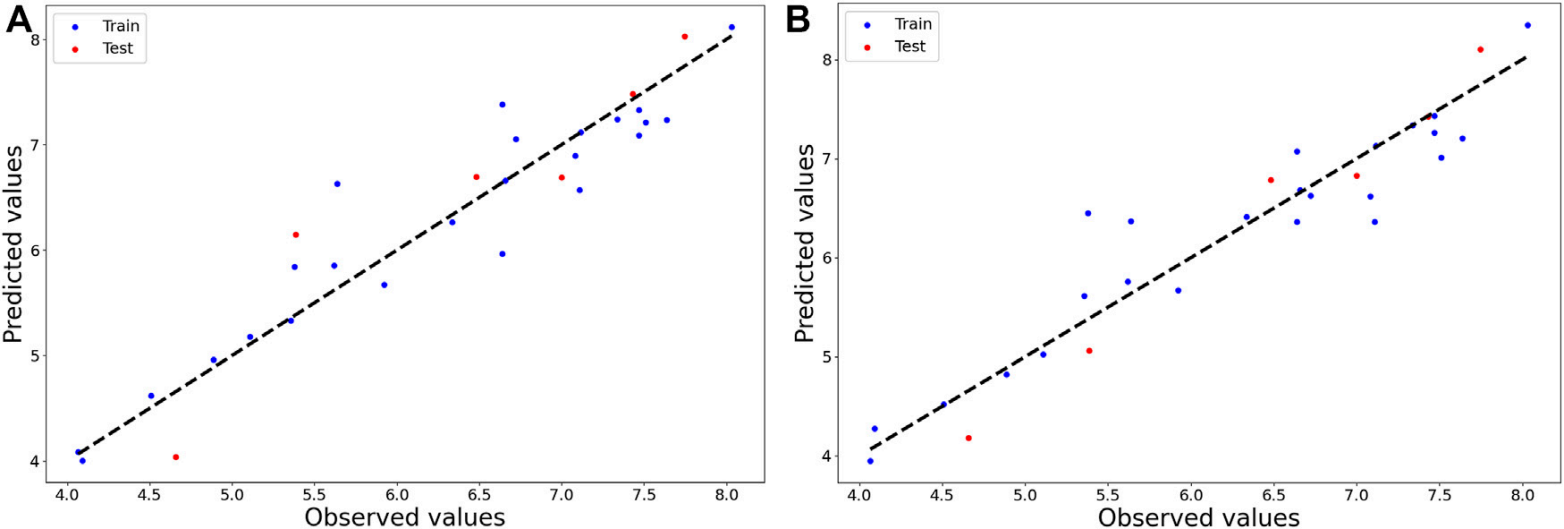
Plots of observed vs. predicted activity of 2D-QSAR **(A)** Model-1 and **(B)** Model-2.

It can be observed from Table 1 that, with increased number as well as complexity of the descriptors, the overall predictivity of the models also improves. GA-MLR clearly generated the most predictive models based on interpretable and 0D-2D descriptors. In case of the former, both the *Q* ^2^_LOO_ and *R* ^2^_Pred_ values (= 0.843 and 0.842, respectively) show that this model is not overfitted, at least. In contrast, the external predictivity increased to a considerable extent when all 2D descriptors are included to set up the model. However, a maximum predictivity was obtained when all descriptors were deployed for model development and in such a case, both feature selection methods yielded highly predictive models through the GA method produced an MLR model that is slightly more predictive than all the SFS-MLR models. Overall, the three developed GA-MLR models were further processed and analyzed. However, before finalizing these models, a test named “5% of CV increment” using the SFS-QSAR tool was performed in order to check whether all five descriptors are truly contributing to their internal validation or not (Halder et al., 2022). To do so, this tool checks if the inclusion of descriptor increases the *Q* ^2^_LOO_ to at least 5% of the existing model. For the current 2D-QSAR modeling, it was more important because the training set comprised 24 data-points and as such, the derived models can contain a maximum of four or five descriptors (as per 1:5 ratio rule that states that the number of descriptors and number of data-points should not exceed 1:5). Interestingly, the model developed with interpretable descriptors retained all five descriptors whereas the model developed with 0D-2D descriptors ended up with three descriptors, which significantly reduced the latter internal predictivity judging from the obtained value for *Q* ^2^_LOO_ (= 0.724). In contrast, the model developed with all (0D-3D) descriptors retained four descriptors but its overall predictivity did not change to a considerable extent as seen from the attained values for *Q* ^2^_LOO_ (= 0.858) and *R* ^*2*^_Pred_ (= 0.923). Therefore, we decided to mainly rely on the five-descriptor model developed with interpretable descriptors (Model-1) and the four-descriptor model developed with all (0D-3D) AlvaDesc descriptors (Mauri, 2020) (Model-2). It is important to note that the four descriptors appearing in Model-2 were also present in the model derived with the scoring functions NMPD or NMGD. The equations pertaining to Model-1 and Model-2 are given below in Table 2 and the observed vs. predicted plots of these models are shown in Figure 1.

**TABLE 2:** Equations and statistical results of the final 2D-QSAR models.

Model	Equation	Statistical results ^a^
1	pEC_50_ = −9.310 (±1.686)	*N*_training_ = 24; *R* ^2^ = 0.898; *R* ^2^_Adj_ = 0.870
	+1.966 (±0.455) B07[C-N]	*F*(18;5) = 31.727; *Q* ^2^_LOO_ = 0.843
	+0.158 (±0.036)Se	MAE = 0.262; *r*_*m*_^2^_ (LOO)_ = 0.783; *∆r*_m_^2^_ (LOO)_ = 0.080; *N*_test_ = 8; *R* ^2^_Pred_ = 0.842; *r*_*m*_^2^_ (test)_ = 0.809
	−0.306 (±0.076) F08[C-C]	*∆r*_*m*_^2^_ (test)_ = 0.095
	+1.278(±0.172) CATS2D_04_DL	
	−0.217(±0.192) CATS2D_02_DL	
2	pEC_50_ = +15.092 (±1.306)	*N*_training_ = 24; *R* ^2^ = 0.889; *R* ^2^_Adj_ = 0.866
	−14.376 (±1.818) GATS6m	*F*(19;4) = 38.14; *Q* ^2^_LOO_ = 0.859
	+0.869(±0.319) B08[C-N]	MAE = 0.272; *r*_*m*_^2^_ (LOO)_ = 0.804; *∆r*_*m*_^2^_ (LOO)_ = 0.068; *N*_test_ = 6; *R* ^2^_Pred_ = 0.923; *r*_*m*_^2^_ (test)_ = 0.790
	–0.173 (±0.076) RDF120p	*∆r*_*m*_^2^_ (test)_ = 0.068
	+0.571 (±0.115) Mor32s	

a *N*_training_, Number of data-points present in the training set; *N*_test_, Number of data-points present in the test set.

So far, we have demonstrated the satisfactory internal and external predictivity of both models but is also important to check the degree of multicollinearity among their variables in addition to their uniqueness. By examining the cross-correlation matrix, we found that Model-1 and Model-2 have a maximum intercollinearity of 0.640 and 0.549, respectively. These values thus suggest that the descriptors of each regression-based model are independent of each other. Next, the calculated ^*c*^ *R*_*p*_^2^ values for Models 1 and 2 were found to be 0.794 and 0.799, respectively, indicating that both models are unique in nature (Ojha and Roy, 2011).

**FIGURE 2:**
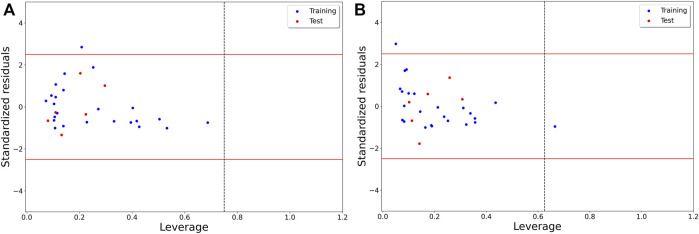
Williams plot describing the applicability domain of **(A)** Model-1 and **(B)** Model-2.

Finally, we assessed the applicability domain of these models by examining the corresponding Williams plots, which are shown in Figure 2. As can be seen, for Model-1, no structural outlier was obtained but one response outlier was found. On the other hand, Model-2 includes one structural outlier and one response outlier. The structural outlier of Model-2 was retained since this model satisfactorily predicted it.

**FIGURE 3:**
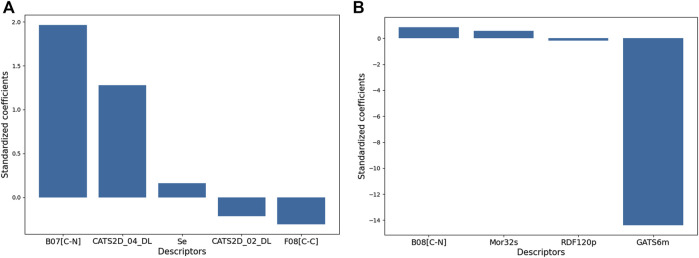
The relative significances of the molecular descriptors appearing in the 2D-QSAR models. The description of each descriptor can be found in the text.

The relative significance of the descriptors of Model 1 and 2 are shown in Figure 3 with respect to their standardized coefficients.

**FIGURE 4:**
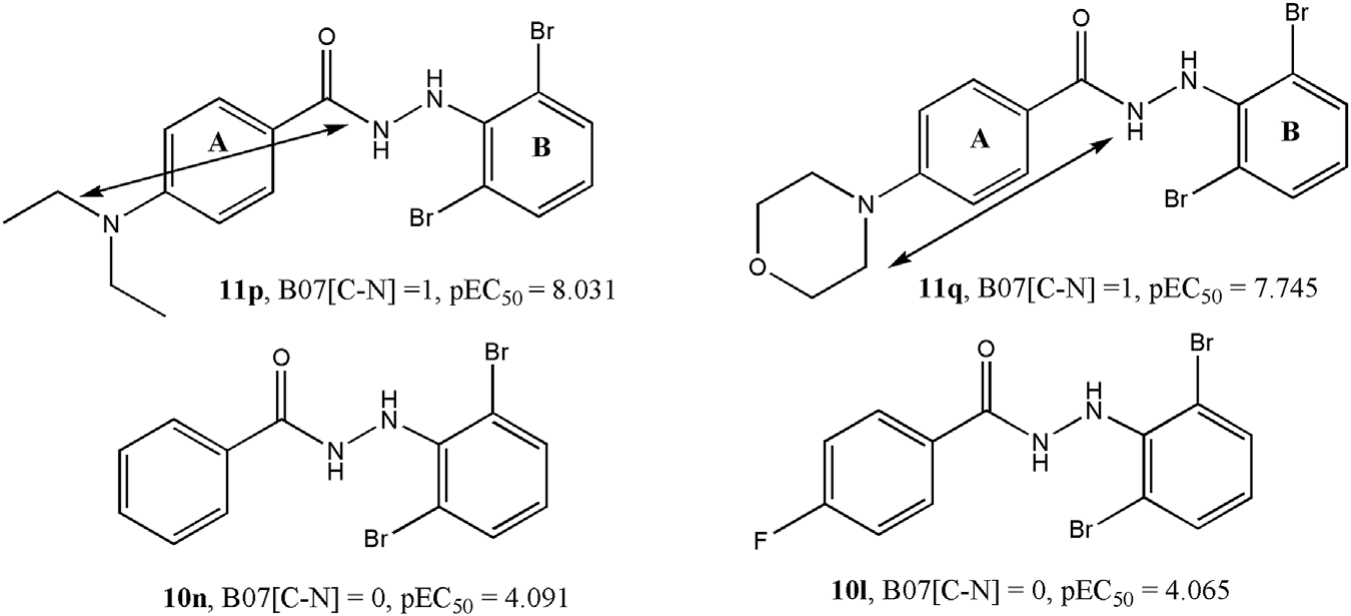
Importance of the B07[C-N] descriptor with respect to the dataset compounds.

Model 1 consisted of five molecular descriptors and among these, B07[C-N] was found to have the maximum contribution. This 2D atom pairs descriptor stands for the presence/absence of C – N at a topological distance of 7. The positive correlation associated with this descriptor suggests that a higher topological distance between carbon and nitrogen atoms may improve the biological activity of these compounds (Todeschini and Consonni, 2000). Now from a mechanistic point of view, this descriptor clearly pinpoints that the presence of carbon-containing residues in the side chain of aromatic ring A improves the activity against the influenza virus. For example, the highly active compounds **11p** and **11q** possess this structural characteristic that is lacking in low active compounds like **10n** and **10l**. From the structures of these compounds, as shown in Figure 4, one can see that steric or hydrophobic interactions with these residues may play important roles in binding of the compounds with the receptors.

**FIGURE 5:**
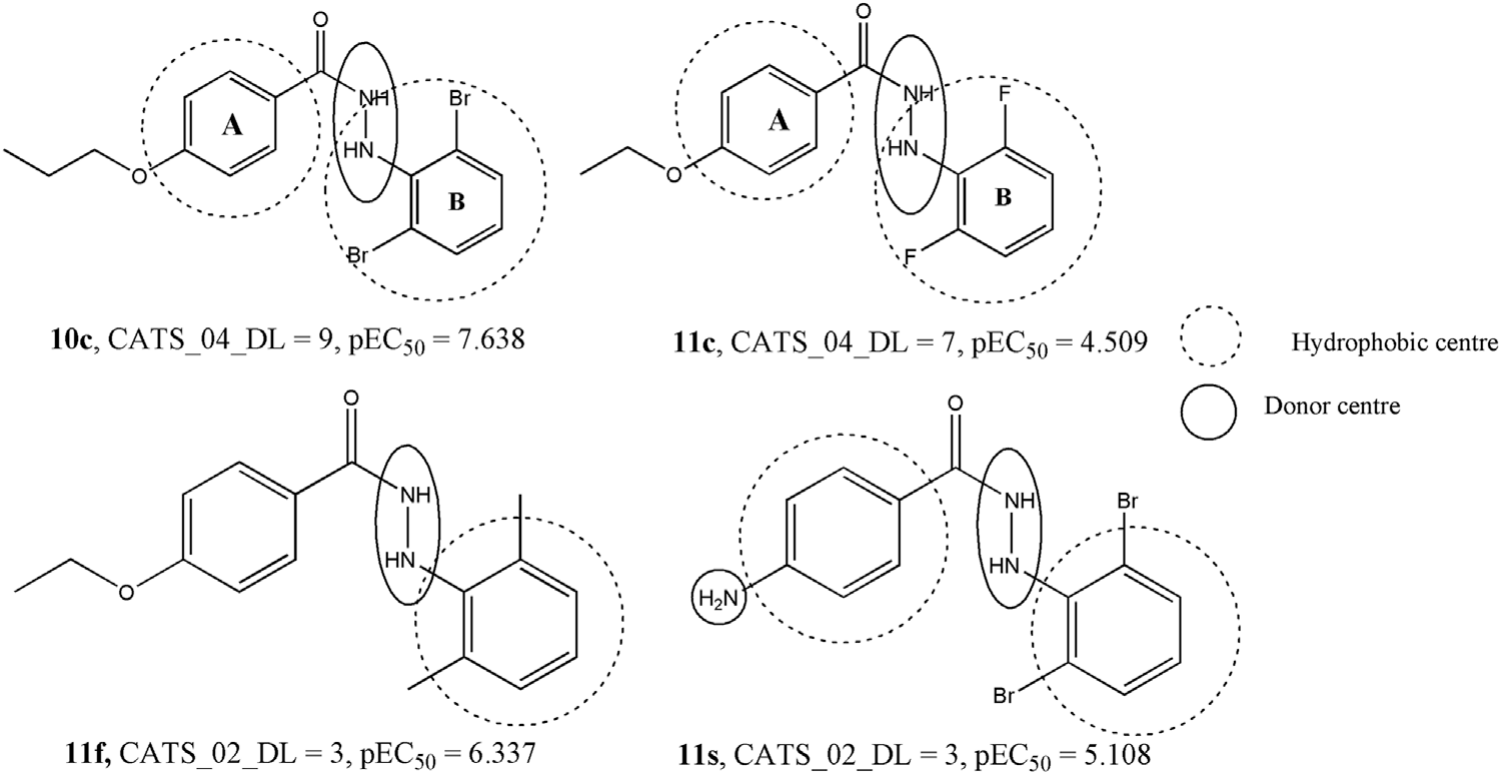
Significance of the CATS2D descriptors with respect to the dataset compounds.

The second most influential descriptor of the model is CATS2D_04_DL, which represent the CATS2D donor-lipophilic at lag (*i.e*., topological distance) 4. CATS2D descriptors are highly useful descriptors that pertain to the topological distance between different pharmacophoric features (Reutlinger et al., 2013). In this case, the hydrogen bond donor and lipophilic features separated at topological distance four are likely to favor higher anti-influenza activity. The value of this descriptor remains consistently high in compounds with high potency whereas its lowest value was observed for **11c**, a compound found to display a low activity against influenza virus. In Figure 5, we compare this structure with one of its closest analogues − *i.e*., compound **10c**, to find if it is actually the substitution of ring B (*cf.* Figure 5) that brings about substantial changes in their biological activities. When the bromine atoms of **10c** are replaced with fluorine, the biological activity drops indicating that the hydrophobicity of ring B plays a crucial role in the interaction of these compounds with the receptor. However, another CATS2D descriptor (*i.e.*, CATS2D_02_DL) was found to have a negative correlation with the biological activity. This indicates that the hydrogen bond donor ability and lipophilicity play important roles in determining the biological activity but their positions in the molecules are also significant as low distances between these pharmacophoric features are detrimental to the biological activity. Some compounds for which high values of the CATS2D_02_DL are found are **11f** and **11s** (Figure 5).

As it is observed from Figure 3, the third contributing feature of the Model-1 is another 2D atom-pair fragment descriptor F08[C-C], which stands for the frequency of C – C at a topological distance of 8. With a negative correlation with pEC_50_, this descriptor indicates that apart from the distance between carbon and nitrogen atom, the distance between two carbon atoms also rules out the biological activity. Electronegativity appears as another important factor since the descriptor named *Se* (sum of the atomic Sanderson electronegativities scaled on the Carbon atom) displayed positive influence on the higher anti-influenza property of these compounds. Even though significance-wise this descriptor comes last in the model, clearly highlighting the importance of electronegativity, which was found to be a major contributor in drug receptor interactions as observed from the 3D-QSAR modeling (described below) (Todeschini and Consonni, 2000).

Model 2 contains four descriptors, two of which belong to the class of 2D descriptors and the remaining two fall under the category of 3D molecular descriptors. Clearly, GATS6m is the most significant descriptor and this 2D autocorrelation descriptor represents the Geary autocorrelation of lag 6 weighted by mass (Todeschini and Consonni, 2000). In autocorrelation descriptors, the molecular structures of the chemical compounds are represented by graphs and the physicochemical properties of their atoms (e.g., mass, volume, electronegativity) are assigned to the vertices of the graph (Hollas, B., 2003). Such descriptors thus depict the distribution of a certain physicochemical property in the topological structure and GATS6m thus represents the distribution of atomic mass at a distance of six bonds in the topological structure of molecule. GATS6m is originated from the Geary coefficient (Geary, R.C., 1954), which is basically a distance-type function, the values of which vary from zero to infinity. Strong autocorrelation produces low values of this index; moreover, positive autocorrelation produces values between 0 and 1, whereas negative autocorrelation generates values larger than 1. A negative correlation between GATS6m and pEC_50_ indicates that the high value of this descriptor is detrimental to biological activity. Interestingly, the 2D-atom pair descriptor B08[C-N] also appears in this model and this descriptor is highly similar to the B07[C-N] descriptor (shown in Figure 4), which was found to be the most significant descriptor of Model-2. Note that both B08[C-N] and B07[C-N] descriptors have positive correlations with the biological activity signifying that the distance between carbon and nitrogen atoms separated with topological distance 7 or 8 plays a crucial role in governing the biological activity of these compounds. The remaining two descriptors of Model-2 suggest that the intrinsic state and polarizability of the compounds are also important contributors to their biological properties. Mor32s, which is the third most significant descriptor of the Model-2, is a 3D-Morse descriptor that stands for signal 32/weighted by the intrinsic-state (Devinyak et al., 2014). The values of 3D-Morse descriptors are extremely sensitive to the starting geometries of the chemical structures as these descriptors originated from the equations used in electron diffraction studies. Apart from Mor32s, RDF120p is another 3D-descriptor that was used to set-up Model-2 and this descriptor stands for the Radial Distribution Function – 120 weighted by polarizability. The values of RDF descriptors largely depend on the interatomic distances (González et al., 2008). Similar to Mor32s, this RDF descriptor depicted a positive correlation with the biological activity. Details of Model-1 and Model-2 including the descriptors of these models and dataset division information can be found in Supplementary Table S1.

The 2D-QSAR models (Model-1 and Model-2) may be generated using the.csv files provided in the Github repository https://github.com/ncordeirfcup/InsilicoModeling_RdRp using web-application https://amit-mlr.herokuapp.com/to check details of the models such as the predicted activities, correlation matrix, plots, *etc*.

### Structure based pharmacophore mapping

**FIGURE 6:**
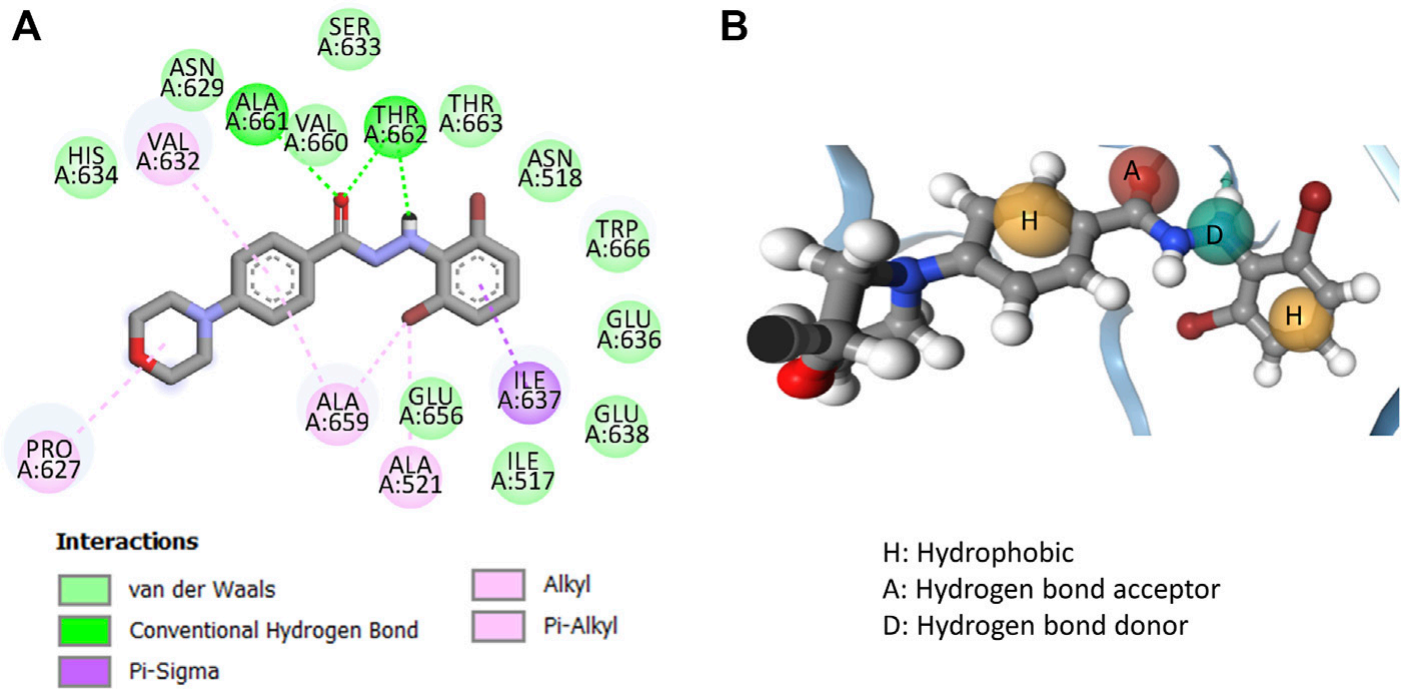
**(A)** Interactions obtained from molecular docking of compound **11q** with the RdRp receptor (PDB: 6QPF) and **(B)** structure-based pharmacophore mapping aligned with **11q**.

In order to further understand how these compounds may actually interact with the receptor (*i.e.*, the RdRp enzyme), one of the most potent compounds (*e.g.*, **11q**) was docked with the B-chain of the RdRp protein of H5N1 (PDB: 6QPF). It is important to note here that Liu et al. previously performed a semi-rigid molecular docking with the same compound against this protein and the blind docking analysis performed by the authors identified the most promising ligand binding site for the ligand (Liu et al., 2022). In the current work, we repeated the blind docking experiment using Autodock Vina and find the same result obtained by Liu et al. (Liu et al., 2022). Therefore, the same binding site was chosen for the present semi-rigid molecular docking with Autodock Vina. The best docked pose (depicted in Figure 6) with a binding score of −7.5 kcal/mol was then selected for constructing the structure-based pharmacophore mapping using the newly developed tool Openpharmacophore. After deriving the pharmacophore, the redundant feature was removed and the final pharmacophore contained four features that are also depicted in Figure 6.

The docked pose of compound **11q** depicted hydrogen bond interactions with Val660 and Thr662. The carbonyl group of the compounds formed hydrogen bind interactions with both Val 660 and Thr662, whereas the secondary amine of hydrazine formed a hydrogen bond interaction with the Thr660 residue of the RdRp protein of H5N1. Major hydrophobic interactions are obtained with the residues Ile637, Ala659, Ala521, Val632 and Pro627. These interactions were found to generate four pharmacophore features in the structure-based pharmacophore mapping and these are two hydrophobic interactions, one hydrogen bond acceptor and one hydrogen bond donor features. Interestingly, even though hydrophobic interactions were reported with the morpholine side chain of **11q**, no feature was generated during structure-based pharmacophore mapping. However, significance of both these docking and pharmacophore model should be established through proper validation. Therefore, the generated structure-based pharmacophore was first used to screen as well as align all dataset compounds (including **11q)**. Save for compound **11c** that appeared as a structural outlier in the 2D-QSAR Model-2, all compounds were embedded into the pharmacophore. The failure of this compound against the pharmacophore model may be justified from the fact that the hydrophobicity of ring B (ring B is shown in Figure 5) was too low as revealed from the 2D-QSAR analysis. Since **11c** was one of the least active compounds in the current dataset, the pharmacophore aligned conformations of the rest of the compounds were used for deriving the 3D-QSAR model that is discussed in the next section.

### 3D-QSAR modeling

In order to further understand how these compounds may interact with the receptor, a 3D-QSAR analysis was performed with the 29 compounds that were properly embedded within the structure-based pharmacophore. It is well known that unlike 2D-QSAR, the 3D-QSAR methodology largely depends on the bioactive conformers of the ligands and their alignment (Verma et al., 2010). The development of the structure-based pharmacophore and alignment of the dataset compounds with this pharmacophore was discussed in the previous section. The aligned conformations were randomly divided into 23 training and six test set compounds (*i.e.*, compounds **10a, 10b, 10m, 10q, 10y** and **11j**, check the Supplementary Table S1). The 3D-QSAR models were generated by employing two techniques – *i.e.*, FFD-SEL and UVE-PLS, as implemented in Open3DQSAR. The statistical quality of the models is depicted in Table 3, from which it may be inferred that the pharmacophore aligned conformations adequately characterize the experimental activity exhibited by the compounds present in the current dataset. Evidently, the UVE-PLS technique is found to be more successful in deducing a more predictive 3D-QSAR model when compared to the FFD-SEL technique. This model was produced with a *Q* ^2^_LOO_ of 0.718 and a *R* ^2^_Pred_ of 0.672. Randomization or scrambling test suggested that the model was indeed unique in nature as the *Q*_s_^2^ value was found to be 0.432 that is considerably lower than all international validation parameters including *Q* ^2^_LMO_. Significantly, these results of 3D-QSAR validate the developed structure-based pharmacophore which in turn justifies the interactions obtained from molecular docking. Therefore, it may be inferred that these aryl benzoyl hydrazide derivatives may actually bind to the binding site proposed earlier by Liu et al. (Liu et al., 2022) and the derived structure-based pharmacophore model may indeed be used to screen and predict the anti-viral activity of these compounds.

**TABLE 3:** Statistical results of 3D-QSAR analysis.

Parameter ^a^	Structure-based alignment		Atom-based alignment	
	FFD-SEL	UVE-PLS	FFD-SEL	UVE-PLS
PC	2	2	3	2
*N*_*training*_	23	23	23	23
*F*-test	224.81	363.96	245.31	256.59
*R* ^2^/SDEC	0.957/0.234	0.973/0.186	0.970/0.180	0.962/0.220
*Q* ^2^_LOO_/SDEP	0.662/0.660	0.718/0.603	0.877/0.397	0.892/0.374
*Q* ^2^_LTO_/SDEP	0.620/0.700	0.685/0.637	0.871/0.407	0.880/0.380
*Q* ^2^_LMO_/SDEP	0.570/0.742	0.637/0.682	0.842/0.448	0.871/0.405
*N*_*Test*_	6	6	6	6
*R* ^2^_Pred_/SDEP	0.672/0.570	0.672/0.587	0.760/0.502	0.749/0.513
*Q*_s_^2^	ND	0.432	ND	0.651

a PC, Number of principal components.

**FIGURE 7:**
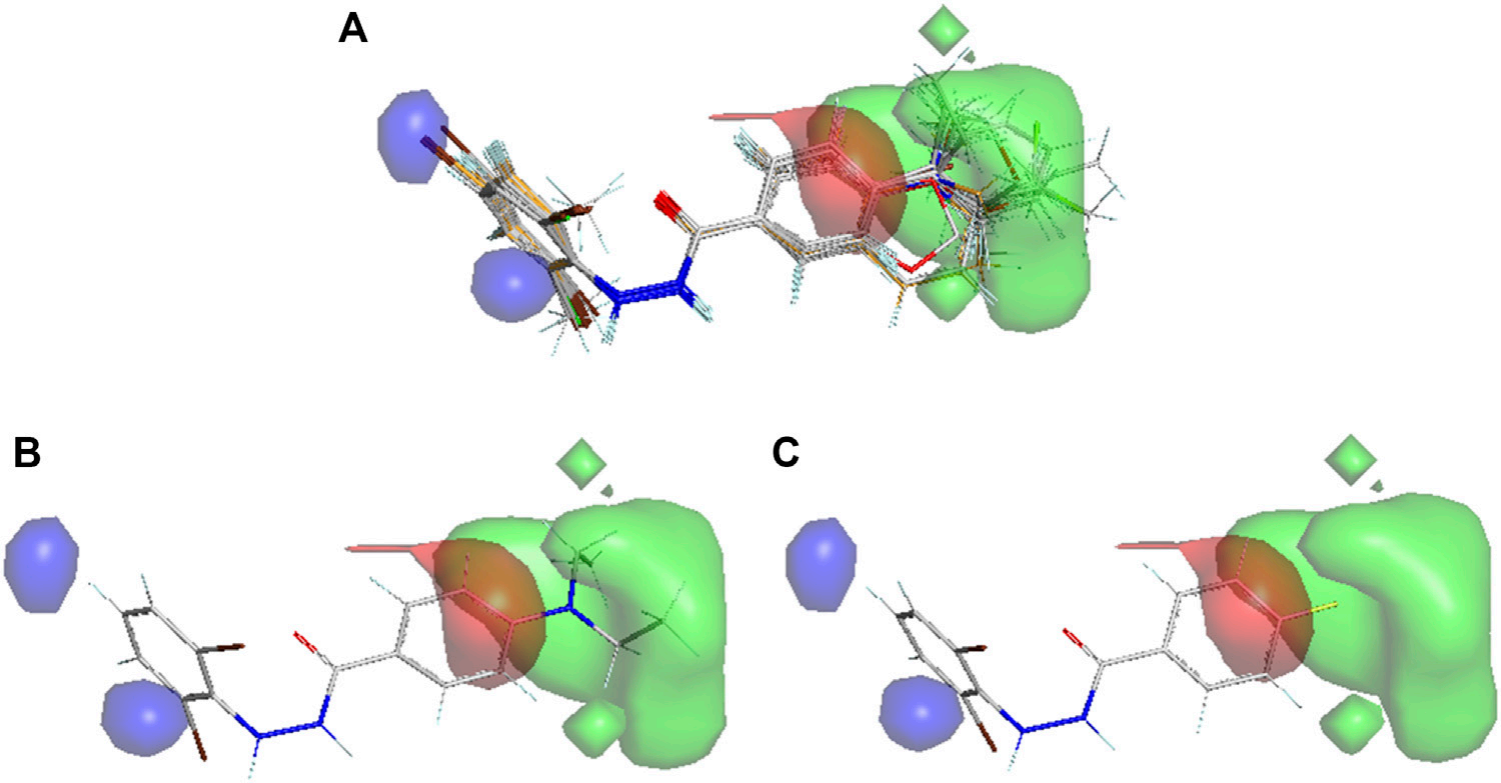
Contour maps obtained from the best 3D-QSAR model (Green: Steric favorable, Blue: Electropositive favorable; Red: Electronegative favorable). **(A)** Aligned conformations of all compounds, **(B)** the best active compound **11p** and **(C)** the least active compound **10l**.

Even though structure-based pharmacophore successfully aligned the conformations of the ligands to generate predictive 3D-QSAR models, we also resorted to an atom-based alignment (or rigid body molecular alignment) of the structures to check if any better 3D-QSAR model may exist or not. The results of 3D-QSAR models developed with atom-based alignment are presented in Table 3, which in turn clearly demonstrate that the statistical results of these models are considerably improved as compared to the models generated from a structure-based alignment. It is however not surprising because structure-based alignment may sometime be less equipped to properly align the structures as compared to unsupervised rigid body alignment (Tosco et al., 2011). The UVE-PLS based 3D-QSAR model produced with structure-based alignment definitely points out the significance of the pharmacophoric features. Nevertheless, on the basis of statistical significance, we decided to rely on the UVE-PLS based 3D-QSAR models developed with atom-based alignment as far as the mechanistic interpretation is concerned. The observed vs. predicted plots of these two UVE-PLS models are shown in Supplementary Figure S1. From Table 3, it is observed that both FFD-SEL and UVE-PLS techniques generated highly predictive models though the later model was finally selected since it was found to be slightly more predictive (on the basis of average *Q* ^2^_LOO_ and *R* ^2^_Pred_) and at the same time, it was generated with a smaller number of components. The electrostatic and steric contour maps of thus the UVE-PLS model with *Q* ^2^_LOO_ of 0.892 and *R* ^*2*^_Pred_ of 0.749 are presented in Figure 7. Randomization test performed with Open3DQSAR generated a *Q*_*s*_^2^ value of 0.651 that was far less than the original *Q* ^2^_LMO_ value depicting that the model was not developed by chance.

The contributions of steric and electrostatic fields found in the best 3D-QSAR models were 0.69 and 0.31, respectively. Therefore, steric effects mainly contributed to the model development. First, the steric positive fields were found near the substituents of aromatic chain A (*cf.* Figure 4) depicting bulky substituents and leading to higher biological activity. This information was also extracted from our 2D-QSAR analysis (Figure 4). As it is observed from Figure 7, the most potent derivative **11p** contains a side chain that is properly inserted into the steric favorable fields, whereas **10l**, being the least biologically active compound, does not contain any side chain to insert into this steric field. However, more importantly, an electropositive unfavorable field exists close to this steric field indicating that substituents in ring A with a negative partial charge or electron rich elements favor higher biological activity. Even though this information is however not reflected conspicuously in the 2D-QSAR analysis, pharmacophore mapping or docking analyses pinpoint some highly active compounds that indeed hold side chains with electronegative atoms, such as oxygen. The aromatic ring B on the other hand, is found to be close to electropositive unfavorable field indicating that positive partial charge or electron deficient elements favor higher potency. This information is however consistent with the docking analysis where this ring engages in π-alkyl or related interactions (such as π- π) that are favored by the reduced electron density of aromatic ring B. This field should also be produced on the basis of the fact that the substitution of higher electronegative fluorine atom with less electronegative atoms (such as bromine) improved the potency of the compounds, as shown in Figure 5.

It is worth mentioning here that we also attempted to develop 3D-QSAR models with docking-based alignment in which each compound was docked at the proposed binding site and the best pose obtained at that binding cavity was selected for 3D-QSAR modeling. However, the docking-based alignment failed to produce predictive models (*see* Supplementary Table S2) as the models constructed with the other two alignment techniques as shown in Table 3.

### Molecular dynamics simulations

**FIGURE 8:**
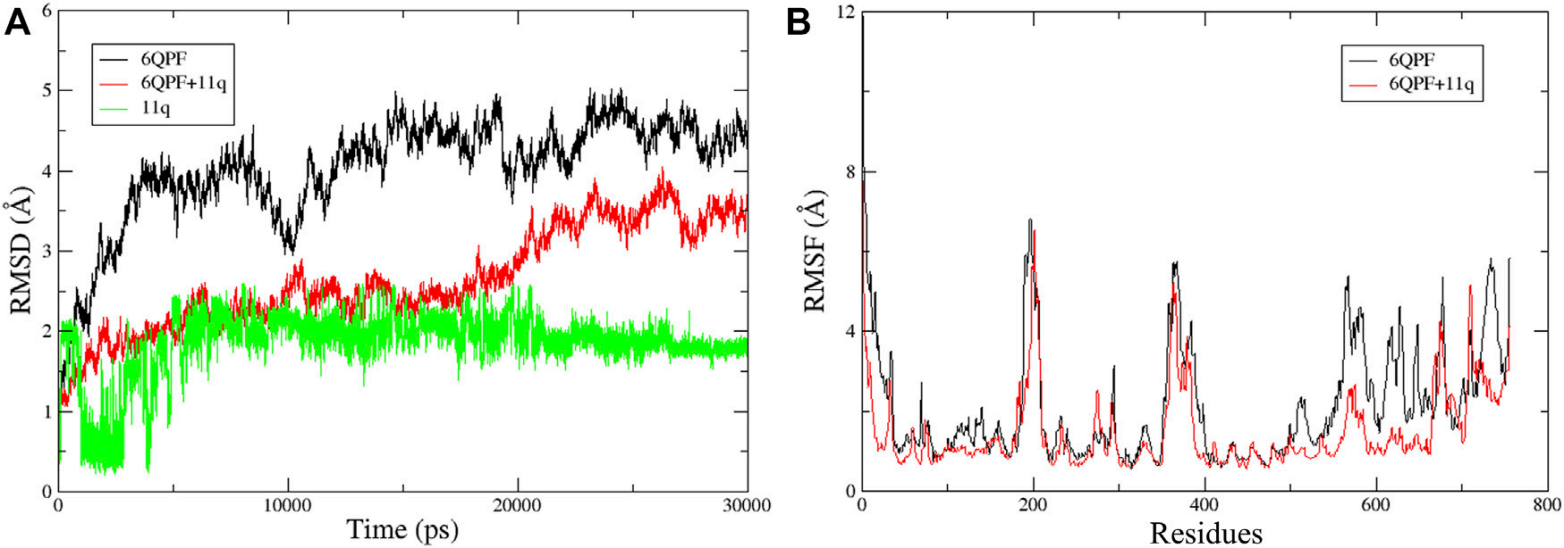
Trajectory analysis results obtained for 30 ns MD simulations: **(A)** The RMSD plots of the apoprotein (6QPF), protein complex (6QPF + **11q**) and docked ligand (**11q**); **(B)** RMSF plots of the apoprotein (6QPF) and protein complex (6QPF + **11q**).

Finally, MD simulations were performed with the docked structure of compound **11q** with the B-chain of RdRp protein to assess the dynamic behavior of the ligand and the complex. At the same time, we wanted to verify if the ligand is stabilized at the proposed binding site and if the proposed structural requirements comply with the MD simulation results or not. It is noteworthy that since the full structure of RdRp is relatively large, only the B-chain of (PB1 domain of the protein) of this protein was investigated by MD. Both the apoenzyme and the complex for MD simulation analysis were used for the comparative analyses. After 30 ns run, the trajectory analysis was done to derive the plots of RMSD and RMSF that are presented in Figure 8. Both apoprotein and complex showed slightly higher RMSD values (most possibly due to the fact that isolated B-chain and not the entire protein was used in the MD simulations as the main objective was to focus on ligand stability and its interactions at the proposed binding cavity) but the RMSD of the complex was found to be reduced than that of the apoenzyme, indicating that binding of the ligand slightly changed the overall fluctuations of the protein. However, the bound ligand became stabilized after 5 ns run indicating that the conformation therein achieved is highly stabilized. From the RMSF plot, it is also visible that as compared to the apoprotein, the complex achieved considerably less fluctuations in the proposed binding region that consists of amino acid residues between 550 and 710, suggesting that the binding of the ligand should have provided stability in this region.

**FIGURE 9:**
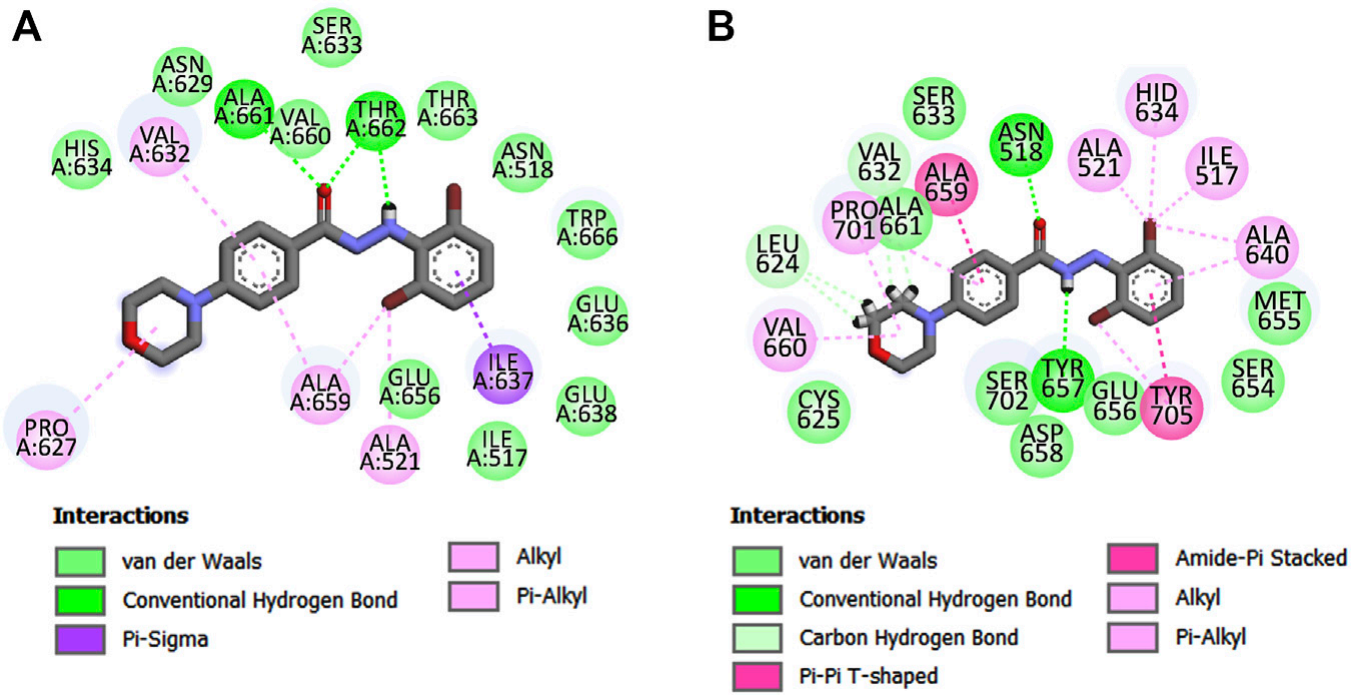
Major ligand-receptor interactions were obtained in **(A)** molecular docking and **(B)** average structure of the protein ligand complex.

To further understand whether the interactions of compound **11q** remained similar to that of its proposed docked pose during the 30 ns run, the average structure of the ligand-bound complex gathered from the MD trajectory was analyzed. The interactions obtained from the average pose are depicted in Figure 9 whereas the average distance and types of interactions are shown in supplementary materials (Supplementary Table S3). The 3D overview of the structures depicting the docked pose and the average structure of the protein-ligand complex is shown in supplementary materials (Supplementary Figure S2).

Noticeably, the interactions obtained after the MD simulation were partially similar to those obtained from the docked pose. For example, the hydrophobic interaction of morpholine residue with Pro627 as well as the hydrophobic interaction of aromatic ring A with Ala659 remained intact. These suggest that the ligand remained in the proposed binding site during the MD simulation. However, the polar interactions with hydrazide moiety as well as the interacting amino acid residues with aromatic chain B (*cf.* Figure 4) altered during the simulations. It is mainly due to the fact that the proposed binding site is adjacent to flexible loops and the rotatable hydrazide moiety of the ligands also imparts flexibility to the aromatic chain B. The change in the conformation of the ligand during 1–7 ns MD simulation is noticeable in the ligand RMSD plot (shown in Figure 8A). Interestingly, these new interactions comply with the mechanistic interpretations obtained from the various *in silico* analyses reported in the current work. For example, the significance of steric residue containing carbon atom attached to an aromatic ring A (*cf.* Figure 4, observed in 2D-QSAR and 3D-QSAR analyses) is established from this pose since this moiety is associated with multiple hydrophobic interactions. Consequently, the importance of pharmacophoric features obtained in the structure-based pharmacophore mapping is also confirmed except for the hydrogen bond donor feature that is shifted from one nitrogen of hydrazide to another. After careful observation, we found that the Tyr657 moiety, which forms hydrogen bond interaction with the ligand may also be accessible to both -NH residues of the hydrazide moiety. Moreover, Tyr705 residue forms π-π interactions with the aromatic ring B, indicating that electron distribution of this ring may be crucial for binding with the receptor and this information complies with the 3D-QSAR model since the negative electrostatic contour map (red colored) was found to be present near the aromatic chain B. The bromine atoms of aromatic ring B also establish hydrophobic interactions justifying the appearance of hydrophobic pharmacophoric feature with this ring. The same justification holds true for the aromatic chain A.

**FIGURE 10:**
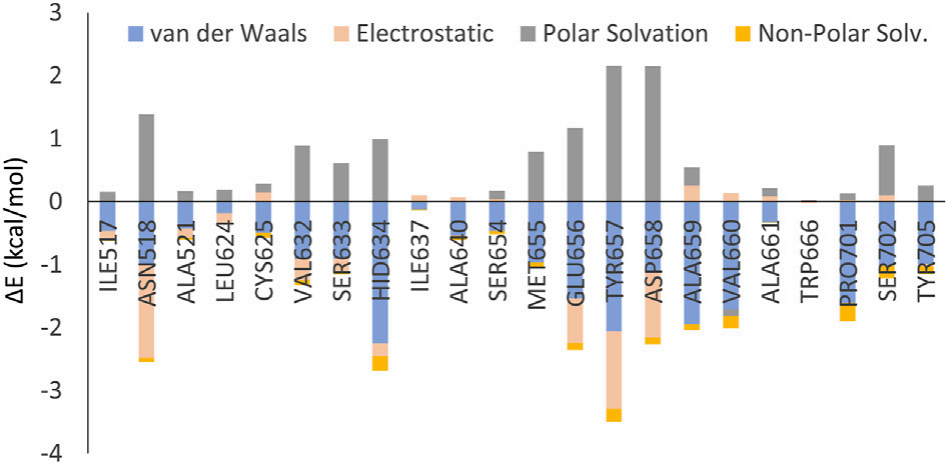
Results of the per-residue decomposition analysis obtained from the MD simulation of the 11q-RdRp complex.

In addition, both MM-GBSA and per-residue decomposition analyses were performed in order to further understand the binding stability and interaction profiles of compound **11q**. The MM-GBSA analysis yielded an enthalpic contribution (∆H) of binding free energy as high as −46.23 kcal/mol (*see* Supplementary Table S4 in supplementary materials). By analyzing its components separately, the van der Waal interaction energy (ΔE_vdW_) was found to be −53.05 kcal/mol whereas the electrostatic energy (ΔE_elec_) −9.70 kcal/mol. As can be seen in Figure 9, the hydrophobic interactions clearly make the major contributions in the ligand-receptor binding. The total solvation free energy (∆G_solv_) was estimated as +16.52 kcal/mol. The results of the per-residue decomposition analysis are depicted in Figure 10. The electrostatic interactions were found to be significant with amino acid residues such as Asn518, Glu656, Tyr657 and Asp658. The van der Waals or hydrophobic interactions were prominent with His634, Ala659, Val660, Pro701, Ser702 and Tyr705. Noticeably, most of these interactions were already revealed by the molecular docking (*see* Figure 9) and the amino acid residues that had high polar solvation energy include Asn518, Glu656, Tyr657 and Asp658. It is worth mentioning here that molecular docking performed by Liu et al. reported halogen bong interactions with one of the bromine atoms of **11q** as one of the most important interactions (Liu et al., 2022). In our MD simulation analyses however, such halogen bond interactions were not found since the per residue decomposition analysis depicted the interactions with bromine atoms as hydrophobic in nature.

## Conclusion

In the present study, various *in silico* analyses have been performed one by one in a systematic manner to understand the structural requirement of aryl benzoyl hydrazide derivatives as anti-viral agents against influenza virus. Within the scope of this work, we attempted to understand possible binding mechanism of these compounds against the RdRp protein of H5N1 of influenza virus. Being one of the most versatile and indispensable enzymes of RNA viruses, this enzyme requires a detailed investigation for the discovery of new antiviral agents. Even though the compounds of the dataset had high structural similarities, these were found to have high variations in their antiviral activity. For that reason, in this work, we tried to systematically explain the variations obtained in the antiviral activity of these compounds with respect to their structural attributes. Liu et al., who reported these novel aryl benzoyl hydrazide derivatives, experimentally discovered the PB1-domain of RdRb protein and identified its possible binding mode using molecular docking. In this work, molecular docking was utilized to construct the structure-based pharmacophore mapping and it was followed by MD simulations to ensure the dynamic stability of the docked ligand at the proposed binding site of the receptor. Moreover, the binding interactions obtained from the MD simulation complied well with other *in silico* approaches such as 2D-QSAR, 3D-QSAR as well as pharmacophore modeling. With high predictive accuracies, these cheminformatic models may be utilized for the design of novel derivatives as anti-influenza virus agents. On the other hand, the proposed binding site of the RdRp protein may be utilized to develop novel compounds with inhibitory potency against this enzyme. Overall, this work provides a wide range of important guidelines to plan the design of new antiviral agents through the inhibition of RdRp enzyme. As per the requirement of a recent health emergency, new antiviral drugs are required to combat against novel corona virus. In this scenario, this study encourages researchers for the development of more promising anti-RNA virus drugs which may be eventually useful for the COVID-19 treatment. More importantly, various *in silico* investigations performed in the current work utilized non-commercial open-access tools and therefore encourages open science.

## Data Availability

The original contributions presented in the study are included in the article/Supplementary Material, further inquiries can be directed to the corresponding authors.
