# Supplementary material for: In silico characterization of aryl benzoyl hydrazide derivatives as potential inhibitors of RdRp enzyme of H5N1 influenza virus
Source: Front Pharmacol. 2022 Sep 26;13:1004255. doi: 10.3389/fphar.2022.1004255 (PMC9548590; doi:10.3389/fphar.2022.1004255)
Supplement: Supplementary file 1 [file DataSheet1.docx]

Supplementary Material

## Supplementary Figures


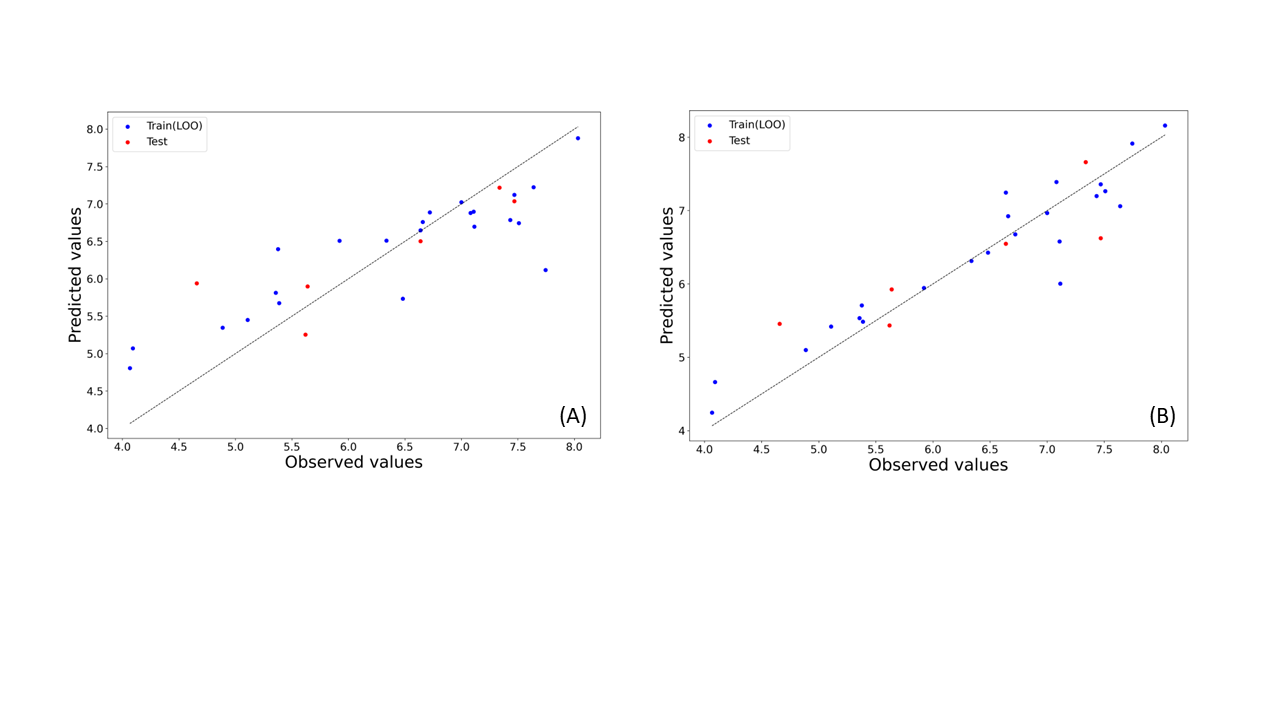


**Supplementary Figure S1.** Observed vs. predicted plots UVE-PLS models of (A) structure-based alignment and (B) atom-based alignment.

## Supplementary Tables

**Supplementary Table S1.** Structures and biological activity of the dataset compounds along with the details (descriptors and dataset division information) of 2D-QSAR Model-1 and Model-2.

| **ID** | **SMILES** | **MDCK**  **EC50 (μM)** | **pIC50** | **Model-1** |  |  |  |  | **Model-2** |  |  |  | **Set** |
| --- | --- | --- | --- | --- | --- | --- | --- | --- | --- | --- | --- | --- | --- |
|  |  |  |  | B07[C-N] | Se | F08[C-C] | CATS2D_04_DL | CATS2D_02_DL | GATS6m | B08[C-N] | RDF120p | Mor32s |  |
| 11p | O=C(NNC1=C(Br)C=CC=C1Br)C2=CC=C(N(CC)CC)C=C2 | 0.0093 | 8.032 | 1 | 42.04 | 8 | 9 | 1 | 0.429 | 1 | 4.914 | -1.044 | Train |
| 11q | O=C(NNC1=C(Br)C=CC=C1Br)C2=CC=C(N3CCOCC3)C=C2 | 0.018 | 7.745 | 1 | 41.49 | 8 | 9 | 1 | 0.497 | 1 | 2.327 | -0.539 | Test |
| 10c | O=C(NNC1=C(Br)C=CC=C1Br)C2=CC=C(OCCC)C=C2 | 0.023 | 7.638 | 1 | 38.39 | 9 | 9 | 1 | 0.539 | 1 | 0.762 | -1.533 | Train |
| 11o | O=C(NNC1=C(Br)C=CC=C1Br)C2=CC=C(N(C)C)C=C2 | 0.031 | 7.509 | 1 | 36.28 | 8 | 9 | 1 | 0.555 | 1 | 2.017 | -1.087 | Train |
| 10b | O=C(NNC1=C(Br)C=CC=C1Br)C2=CC=C(OCC)C=C2 | 0.034 | 7.469 | 1 | 35.5 | 8 | 9 | 1 | 0.538 | 1 | 0.539 | -1.514 | Train |
| 10f | O=C(NNC1=C(Br)C=CC=C1Br)C2=CC=C(OCC(F)(F)F)C=C2 | 0.034 | 7.469 | 1 | 37.04 | 8 | 9 | 1 | 0.594 | 1 | 0.452 | 0.148 | Train |
| 10g | O=C(NNC1=C(Br)C=CC=C1Br)C2=CC=C(C(C)(C)C)C=C2 | 0.037 | 7.432 | 1 | 39.94 | 9 | 9 | 1 | 0.521 | 1 | 3.571 | -0.757 | Test |
| 10y | O=C(NNC1=C(Cl)C=CC=C1Cl)C2=CC=C(N(C)C)C=C2 | 0.046 | 7.337 | 1 | 36.47 | 8 | 9 | 1 | 0.489 | 1 | 2.524 | -2.025 | Train |
| 10u | O=C(NNC1=C(Cl)C=CC=C1Cl)C2=CC=C(OCC)C=C2 | 0.077 | 7.114 | 1 | 35.69 | 8 | 9 | 1 | 0.522 | 1 | 0.542 | -2.168 | Train |
| 10e | O=C(NNC1=C(Br)C=CC=C1Br)C2=CC=C(CC)C=C2 | 0.078 | 7.108 | 1 | 34.17 | 9 | 9 | 1 | 0.614 | 1 | 1.104 | -1.022 | Train |
| 10r | O=C(NNC1=C(Br)C=CC=C1Br)C2=CC=C(C3=CC=CC=C3)C=C2 | 0.083 | 7.081 | 1 | 38.17 | 10 | 9 | 1 | 0.545 | 1 | 2.179 | -1.979 | Train |
| 11b | O=C(NNC1=C(C)C=CC=C1Cl)C2=CC=C(OCC)C=C2 | 0.1 | 7 | 1 | 38.25 | 10 | 9 | 2 | 0.545 | 1 | 0.64 | -2.071 | Test |
| 10x | O=C(NNC1=C(Cl)C=CC=C1Cl)C2=CC=C(CCC)C=C2 | 0.19 | 6.721 | 1 | 37.25 | 9 | 9 | 1 | 0.545 | 1 | 2.468 | -1.876 | Train |
| 11g | O=C(NNC1=C(C)C=CC=C1C)C2=CC=C(C(C)(C)C)C=C2 | 0.22 | 6.658 | 1 | 45.25 | 13 | 9 | 3 | 0.544 | 1 | 4.098 | -1.316 | Train |
| 10d | O=C(NNC1=C(Br)C=CC=C1Br)C2=CC=C(OCCCC)C=C2 | 0.23 | 6.638 | 1 | 36.51 | 8 | 8 | 1 | 0.607 | 1 | 1.691 | -1.013 | Train |
| 11j | O=C(NNC1=C(Br)C=C(Br)C=C1Br)C2=CC=C(N(C)C)C=C2 | 0.23 | 6.638 | 1 | 41.27 | 10 | 9 | 1 | 0.55 | 1 | 0.835 | -1.46 | Train |
| 10k | O=C(NNC1=C(Br)C=CC=C1Br)C2=CC=C(C(OC)=O)C=C2 | 0.33 | 6.481 | 1 | 34.95 | 9 | 9 | 1 | 0.579 | 1 | 2.666 | -0.696 | Test |
| 11f | O=C(NNC1=C(C)C=CC=C1C)C2=CC=C(OCC)C=C2 | 0.46 | 6.337 | 1 | 40.81 | 12 | 9 | 3 | 0.584 | 1 | 0.619 | -1.841 | Train |
| 10p | O=C(NNC1=C(Br)C=CC=C1Br)C2=CC(C=CC=C3)=C3C=C2 | 1.2 | 5.921 | 1 | 34.29 | 12 | 9 | 1 | 0.614 | 1 | 4.11 | -1.318 | Train |
| 10a | O=C(NNC1=C(Br)C=CC=C1Br)C2=CC=C(OC)C=C2 | 2.3 | 5.638 | 1 | 32.62 | 8 | 9 | 1 | 0.607 | 1 | 0.671 | -1.328 | Train |
| 10q | O=C(NNC1=C(Br)C=CC=C1Br)C2=CC(C=CC=N3)=C3C=C2 | 2.4 | 5.62 | 1 | 33.51 | 11 | 9 | 1 | 0.611 | 1 | 3.692 | -1.37 | Train |
| 10w | O=C(NNC1=C(Cl)C=CC=C1Cl)C2=CC=C(C)C=C2 | 4.1 | 5.387 | 1 | 31.48 | 9 | 9 | 1 | 0.605 | 0 | 0.538 | -2.162 | Test |
| 11h | O=C(NNC1=C(Br)C=C(Br)C=C1Br)C2=CC=C(OCC)C=C2 | 4.2 | 5.377 | 1 | 35.73 | 8 | 8 | 1 | 0.585 | 1 | 1.643 | -1.435 | Train |
| 11i | O=C(NNC1=C(Br)C=C(Br)C=C1Br)C2=CC=C(CC)C=C2 | 4.4 | 5.357 | 1 | 34.4 | 9 | 8 | 1 | 0.673 | 1 | 0.895 | -0.908 | Train |
| 11s | O=C(NNC1=C(Br)C=CC=C1Br)C2=CC=C(N)C=C2 | 7.8 | 5.108 | 0 | 30.51 | 8 | 10 | 3 | 0.692 | 1 | 0.169 | -1.685 | Train |
| 10s | O=C(NNC1=C(Br)C=CC=C1Br)C2=CC(OCO3)=C3C=C2 | 13 | 4.886 | 1 | 32.06 | 9 | 8 | 1 | 0.658 | 0 | 0.955 | -1.124 | Train |
| 10m | O=C(NNC1=C(Br)C=CC=C1Br)C2=CC=C(Br)C=C2 | 22 | 4.658 | 0 | 28.64 | 8 | 9 | 1 | 0.707 | 0 | 0.003 | -1.298 | Test |
| 11c | O=C(NNC1=C(F)C=CC=C1F)C2=CC=C(OCC)C=C2 | 31 | 4.509 | 1 | 36.07 | 8 | 7 | 1 | 0.611 | 1 | 0.547 | -4.487 | Train |
| 10n | O=C(NNC1=C(Br)C=CC=C1Br)C2=CC=CC=C2 | 81 | 4.092 | 0 | 28.41 | 8 | 9 | 1 | 0.695 | 0 | 0.001 | -1.452 | Train |
| 10l | O=C(NNC1=C(Br)C=CC=C1Br)C2=CC=C(F)C=C2 | 86 | 4.066 | 0 | 28.92 | 8 | 9 | 1 | 0.69 | 0 | 0.029 | -2.126 | Train |

**Supplementary Table S2.** Statistical results of 3D-QSAR analysis performed with docking based alignment.

| **Parameter ^a^** | **Docking-based alignment** | |
| --- | --- | --- |
|  | **FFD-SEL** | **UVE-PLS** |
| PC | 3 | 5 |
| *N_Training_* | 23 | 23 |
| F-test | 28.437 | 51.897 |
| *R*^2^/SDEC | 0.818/0.484 | 0.938/0.281 |
| *Q*^2^_LOO_/SDEP | 0.348/0.916 | 0.600/0.718 |
| *Q*^2^_LTO_ /SDEP | 0.288/0.958 | 0.555/0.757 |
| *Q*^2^_LMO_/SDEP | 0.187/1.020 | 0.480/0.813 |
| *N_Test_* | 6 | 6 |
| *R*^2^_Pred_/SDEP | 0.301/0.856 | 0.086/0.979 |
| *Q*_s_^2^ | ND | ND |

**Supplementary Table S3.** Average distances of the ligand-receptor interactions obtained from MD simulations.

| **Amino acid residue** | **Distance (Å)** | | **Type of interaction** |  |
| --- | --- | --- | --- | --- |
| Tyr657 | 2.2 | H-bonding | | |
| Asn518 | 2.36 | H-bonding | | |
| Tyr705 | 3.65 | pi-pi | | |
| Ala521 | 3.65 | Hydrophobic | | |
| Ile517 | 4.17 | Hydrophobic | | |
| His634 | 4.23 | Hydrophobic | | |
| Ala640 | 4.23 | Hydrophobic | | |
| Ala659 | 4.72 | Hydrophobic | | |
| Pro701 | 4.62 | Hydrophobic | | |
| Val660 | 5.31 | Hydrophobic | | |
| Val632 | 3.05 | Hydrophobic | | |

**Supplementary Table S4.** Calculated enthalpic contribution of binding free energies [ΔG_bind_(T)] of the complex. The energy components are in kcal/mole

| **Complexes** | **ΔE_vdW_** | **ΔE_elec_** | **ΔG_gas_** | **ΔG_polar_** | **ΔG_nonpolar_** | **ΔG_solv_** | **ΔG_bind_(T)** |
| --- | --- | --- | --- | --- | --- | --- | --- |
| 11q-6QPF | −53.05 | −9.70 | −62.76 | +21.93 | −5.40 | +16.52 | −46.23 |

## Supplementary Text

**Supplementary Text S1.** Structural similarity analyses with dataset compounds.

In order to check how much structural similarity exists among the dataset compounds, a newly developed *in house* tool named SIMSEARCH (<https://github.com/ncordeirfcup/SIMSEARCH>) (Halder and Cordeiro, 2021) was explored. In this tool, the most potent compound of the dataset (i.e., **11p**) was used as a query compound whereas the remaining dataset compound was used as target dataset. SIMSEARCH calculates the Euclidean distance between each query and target dataset compound with respect to fingerprints calculated with these chemical structures and in the current work, FCFP4 (Feature-class fingerprint of diameter 4, circular) fingerprint was chosen for quantitative similarity analyses (O'Boyle and Sayle, 2016). The results are presented in Table ST1.

**Table ST1.** The results of quantitative similarity analysis.

| **Query Cpd** | | **Query** | **Name** | **Target** | **Similarity** |
| --- | --- | --- | --- | --- | --- |
| 11p | CCN(CC)c1ccc(C(=O)NNc2c(Br)cccc2Br)cc1 | | 11q | O=C(NNc1c(Br)cccc1Br)c1ccc(N2CCOCC2)cc1 | 0.822 |
| 11p | CCN(CC)c1ccc(C(=O)NNc2c(Br)cccc2Br)cc1 | | 11o | CN(C)c1ccc(C(=O)NNc2c(Br)cccc2Br)cc1 | 0.821 |
| 11p | CCN(CC)c1ccc(C(=O)NNc2c(Br)cccc2Br)cc1 | | 10y | CN(C)c1ccc(C(=O)NNc2c(Cl)cccc2Cl)cc1 | 0.821 |
| 11p | CCN(CC)c1ccc(C(=O)NNc2c(Br)cccc2Br)cc1 | | 10b | CCOc1ccc(C(=O)NNc2c(Br)cccc2Br)cc1 | 0.809 |
| 11p | CCN(CC)c1ccc(C(=O)NNc2c(Br)cccc2Br)cc1 | | 10u | CCOc1ccc(C(=O)NNc2c(Cl)cccc2Cl)cc1 | 0.809 |
| 11p | CCN(CC)c1ccc(C(=O)NNc2c(Br)cccc2Br)cc1 | | 11c | CCOc1ccc(C(=O)NNc2c(F)cccc2F)cc1 | 0.809 |
| 11p | CCN(CC)c1ccc(C(=O)NNc2c(Br)cccc2Br)cc1 | | 10c | CCCOc1ccc(C(=O)NNc2c(Br)cccc2Br)cc1 | 0.800 |
| 11p | CCN(CC)c1ccc(C(=O)NNc2c(Br)cccc2Br)cc1 | | 10d | CCCCOc1ccc(C(=O)NNc2c(Br)cccc2Br)cc1 | 0.792 |
| 11p | CCN(CC)c1ccc(C(=O)NNc2c(Br)cccc2Br)cc1 | | 10a | COc1ccc(C(=O)NNc2c(Br)cccc2Br)cc1 | 0.765 |
| 11p | CCN(CC)c1ccc(C(=O)NNc2c(Br)cccc2Br)cc1 | | 10g | CC(C)(C)c1ccc(C(=O)NNc2c(Br)cccc2Br)cc1 | 0.746 |
| 11p | CCN(CC)c1ccc(C(=O)NNc2c(Br)cccc2Br)cc1 | | 10e | CCc1ccc(C(=O)NNc2c(Br)cccc2Br)cc1 | 0.714 |
| 11p | CCN(CC)c1ccc(C(=O)NNc2c(Br)cccc2Br)cc1 | | 10f | O=C(NNc1c(Br)cccc1Br)c1ccc(OCC(F)(F)F)cc1 | 0.711 |
| 11p | CCN(CC)c1ccc(C(=O)NNc2c(Br)cccc2Br)cc1 | | 10x | CCCc1ccc(C(=O)NNc2c(Cl)cccc2Cl)cc1 | 0.708 |
| 11p | CCN(CC)c1ccc(C(=O)NNc2c(Br)cccc2Br)cc1 | | 11j | CN(C)c1ccc(C(=O)NNc2c(Br)cc(Br)cc2Br)cc1 | 0.699 |
| 11p | CCN(CC)c1ccc(C(=O)NNc2c(Br)cccc2Br)cc1 | | 10w | Cc1ccc(C(=O)NNc2c(Cl)cccc2Cl)cc1 | 0.696 |
| 11p | CCN(CC)c1ccc(C(=O)NNc2c(Br)cccc2Br)cc1 | | 10n | O=C(NNc1c(Br)cccc1Br)c1ccccc1 | 0.691 |
| 11p | CCN(CC)c1ccc(C(=O)NNc2c(Br)cccc2Br)cc1 | | 11h | CCOc1ccc(C(=O)NNc2c(Br)cc(Br)cc2Br)cc1 | 0.689 |
| 11p | CCN(CC)c1ccc(C(=O)NNc2c(Br)cccc2Br)cc1 | | 11b | CCOc1ccc(C(=O)NNc2c(C)cccc2Cl)cc1 | 0.685 |
| 11p | CCN(CC)c1ccc(C(=O)NNc2c(Br)cccc2Br)cc1 | | 11s | Nc1ccc(C(=O)NNc2c(Br)cccc2Br)cc1 | 0.671 |
| 11p | CCN(CC)c1ccc(C(=O)NNc2c(Br)cccc2Br)cc1 | | 10m | O=C(NNc1c(Br)cccc1Br)c1ccc(Br)cc1 | 0.671 |
| 11p | CCN(CC)c1ccc(C(=O)NNc2c(Br)cccc2Br)cc1 | | 10l | O=C(NNc1c(Br)cccc1Br)c1ccc(F)cc1 | 0.671 |
| 11p | CCN(CC)c1ccc(C(=O)NNc2c(Br)cccc2Br)cc1 | | 10k | COC(=O)c1ccc(C(=O)NNc2c(Br)cccc2Br)cc1 | 0.667 |
| 11p | CCN(CC)c1ccc(C(=O)NNc2c(Br)cccc2Br)cc1 | | 10s | O=C(NNc1c(Br)cccc1Br)c1ccc2c(c1)OCO2 | 0.653 |
| 11p | CCN(CC)c1ccc(C(=O)NNc2c(Br)cccc2Br)cc1 | | 11i | CCc1ccc(C(=O)NNc2c(Br)cc(Br)cc2Br)cc1 | 0.605 |
| 11p | CCN(CC)c1ccc(C(=O)NNc2c(Br)cccc2Br)cc1 | | 11f | CCOc1ccc(C(=O)NNc2c(C)cccc2C)cc1 | 0.597 |
| 11p | CCN(CC)c1ccc(C(=O)NNc2c(Br)cccc2Br)cc1 | | 10p | O=C(NNc1c(Br)cccc1Br)c1ccc2ccccc2c1 | 0.568 |
| 11p | CCN(CC)c1ccc(C(=O)NNc2c(Br)cccc2Br)cc1 | | 10q | O=C(NNc1c(Br)cccc1Br)c1ccc2ncccc2c1 | 0.568 |
| 11p | CCN(CC)c1ccc(C(=O)NNc2c(Br)cccc2Br)cc1 | | 10r | O=C(NNc1c(Br)cccc1Br)c1ccc(-c2ccccc2)cc1 | 0.547 |
| 11p | CCN(CC)c1ccc(C(=O)NNc2c(Br)cccc2Br)cc1 | | 11g | Cc1cccc(C)c1NNC(=O)c1ccc(C(C)(C)C)cc1 | 0.512 |

The range of structural similarity was found as 0.512-0.822 with a median value of 0.696. Therefore, it may be inferred from the results that a high structural similarity exists among the dataset compounds.

**References**

Halder, A. K., Cordeiro, M. N. D. S. (2021) Multi-target in silico prediction of inhibitors for mitogen-activated protein kinase-interacting kinases. *Biomolecules* 11(11), 1670. https://doi.org/10.3390/biom11111670

O'Boyle, N. M., Sayle, R. A. (2016) Comparing structural fingerprints using a literature-based similarity benchmark. *Journal of Cheminformatics*, 8, 36. doi: 10.1186/s13321-016-0148-0.
